# Protective and Non-Protective Factors of Mental Health Distress in the United States during the COVID-19 Pandemic: A Systematic Review

**DOI:** 10.3390/medicina57121377

**Published:** 2021-12-17

**Authors:** Cristian Lieneck, Michele Bosworth, Eric Weaver, Katharine Heinemann, Janki Patel

**Affiliations:** 1 School of Health Administration, Texas State University, San Marcos, TX 78666, USA; kah357@txstate.edu (K.H.); jdp285@txstate.edu (J.P.); 2 The Center for Population Health, Analytics, Quality Advancement in The School of Community and Rural Health, The University of Texas at Tyler, Tyler, TX 75799, USA; michele.bosworth@uthct.edu; 3 Institute for Advancing Health Value, Western Governors University, Salt Lake City, UT 84107, USA; eric.weaver@wgu.edu

**Keywords:** mental health, behavioral health, assessment, telehealth, COVID-19

## Abstract

*Background and objectives:* Health care organizations continue to respond to the COVID-19 global pandemic and an ongoing array of related mental health concerns. These pandemic-related challenges continue to be experienced by both the U.S. population and those abroad. *Materials and methods:* This systematic review queried three research databases to identify applicable studies related to protective and non-protective factors of mental health distress experienced during the pandemic within the United States. *Results:* Three primary factors were identified as protective factors, potentially helping to moderate the incidence of mental distress during the pandemic: demographics, personal support/self-care resources, and income/financial concerns. Researchers also identified these same three constructs of non-protective factors of mental health distress, as well as two additional variables: health/social status and general knowledge/government mistrust. *Conclusions:* This systematic review has identified protective and non-protective factors of mental health distress experienced in the United States during the COVID-19 pandemic (to date) that can further assist medical providers in the U.S. and beyond as the pandemic and related mental health concerns continue at a global level.

## 1. Introduction

### 1.1. Rationale

The coronavirus SARS-CoV-2 (COVID-19) pandemic is not only a global entity, but is also an equal opportunity pathogen. The physiologic and physical symptoms incurred by virus victims have a wide spectral berth, from asymptomatic to fatal. The rapid onset and dissemination of “the virus” to global proportions took the world’s population by surprise in early 2020. While the global medical community raced for treatment and prevention, the virus was also claiming a sinister impact on mental health, especially in the United States. According to the Center for Disease Control and Prevention (CDC), in June of 2020 (just a few months after the pandemic onset) “40% of US adults reported struggling with mental health or substance abuse”; a three-fold increase compared to 2019 data. Symptoms of mental health concerns included depression, anxiety, suicidal thoughts, stress-related disorder, and substance abuse [1]. Although, an increase in mental health issues occurred, the literature reveals that different population characteristics may have proven to be protective or non-protective. Thus, this review focuses on identifying those key factors categorically.

### 1.2. Objectives

The research team’s overall intent was to investigate underlying themes (constructs) surrounding influences upon the U.S. population’s mental health distress as experienced during the COVID-19 pandemic to date. Both protective and non-protective (facilitator and barrier) constructs were investigated to identify factors contributing to and assisting with guarding against mental health distress and related mental illnesses. The research team’s focus was to evaluate such observations in the literature, code/classify both protective and non-protective factors having an influence on the mental health distress independent variable, and to disseminate research findings to further assist with mental health care and resources provided in an ongoing manner during the pandemic.

The research team hypothesized potential protective factors as experienced themselves throughout the pandemic while living in the U.S., as well as non-protective factors in this regard. These experiences and initial queries into the literature became the impetus for the study, while no pre-identification of any specific protective and/or non-protective mental health/distress constructs were delineated to preserve the integrity of the review. The team also recognized that the duration of the pandemic with additional COVID-19 variants could potentially influence construct identification during limited time periods within the past 1.5 years. Public health policies at local, state, and national levels, as well as economic changes throughout the review period could also affect protective and non-protective factors of mental health distress.

## 2. Materials and Methods

### 2.1. Eligibility Criteria

To be included in the review, studies had to be focused on mental health distress and/or illness and be specifically related and/or occurring during the COVID-19 pandemic. As a result, articles had to be published between 1 March 2020 through 25 May 2021; however, a 2022 search end date was available and utilized by the Elton B. Stephens company (EBSCO) search engine to ensure that all most-recent publications were included in the search at the time of database query. Articles had to be published in quality peer-reviewed and/or academic journals, written in English, and identified with U.S. geographic indicator by EBSCO. While some articles identified included an evaluation of patient outcomes with regard to mental health distress treatment and follow-up care provisions, this was not a required criterion to be included in the review.

### 2.2. Information Sources

The review queried three academic research databases: Academic Search Complete, Complementary Index, and MEDLINE Complete. These three databases were chosen for the review because they yielded the highest frequency of results upon controlling for potential duplicates, as identified by the EBSCO search engine. The search was conducted from May 10, 2021 through May 25, 2021.

### 2.3. Search

Databases selected for the review focused on those that yielded the highest initial search results. The research team developed an aggressive search string with Boolean operators that identified the highest initial database results for the study topic. The National Library of Medicine’s controlled vocabulary thesaurus—Medical Subject Headings (or MeSH)—was used to identify key terms for each of the search variables. After multiple iterations of search queries with various Boolean operators, a final review sample was identified. The final search string identified by the researchers was: (“mental health service*” OR “mental hygiene service*” OR “mental health” OR mental hygiene”) AND (“COVID-19” OR “coronavirus” OR “2019-ncov”) AND (“telemedicine” OR “telehealth” OR “telecare” OR “telecommunication” OR “online” OR “virtual”) AND (“assessment tools” OR “assessment method” OR “assessing”).

The search criteria included an aggressive article publication date range in order to specifically identify mental health assessments conducted using telehealth resources during the COVID-19 global pandemic. Additional search criteria included English-only, peer-reviewed and/or academic journals only, and U.S. geographic location only. These exclusion criteria were executed by using the EBSCO research database filtering criteria options and were then later verified during the full-article review process. While the research team acknowledges the importance of a global perspective on the implementation of telehealth during the pandemic, this review initiative was intended to specifically investigate inherent, U.S.-specific characteristics of mental health assessments conducted via telehealth during the global pandemic.

### 2.4. Initial Study Selection

Preferred Reporting Items for Systematic Reviews and Meta-Analysis (PRISMA) guided this review initiative, and the review was registered on PROSPERO. All researchers participated in the initial database search, which included any/all articles identified by the initial search criteria. The research team are all from various research/academic institutions, and, therefore, the use of full-text as a search criteria was not utilized in the initial database search. Therefore, a maximum number of initial articles was identified by the research team. Capitalizing on each research team members’ home institution (university) research database access privileges, the full text of all identified articles was able to be accessed using a collective process.

A MS Excel spreadsheet was used to document the review team’s efforts in reviewing the literature, division of work, identifying and recording identified themes in the sample, and related sub-theme affinity commentary between team members. Multiple methods were utilized in this initial review, including abstract screening, full text article review, and also review of the initial sample articles’ literature cited/reference sections. The team conducted multiple webinar sessions to collaborate on the literature review matrix findings, and no content and/or underlying theme disagreements were experienced at this stage of the review.

## 3. Results

### 3.1. Study Selection/Exclusion

**Figure 1:**
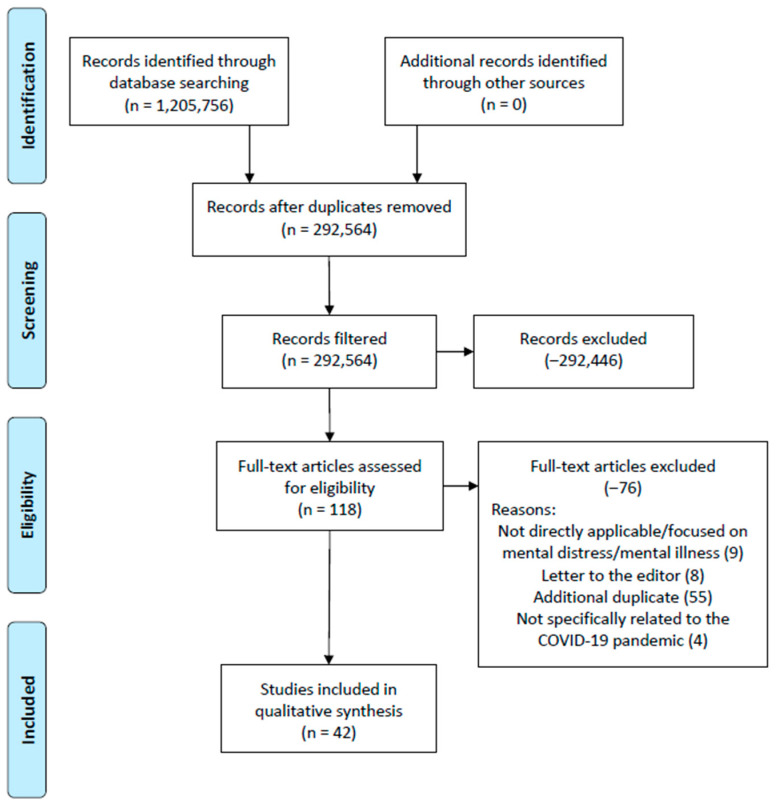
Preferred reporting items for systematic reviews and meta-analysis (PRISMA) figure that demonstrates the PRISMA-guided study selection process.

The study selection and subsequent article exclusion process is shown in Figure 1. While over 1.2 million articles were initially identified in the initial database search using the search string developed using MeSH key terms, the exclusion process removed a significant amount of duplicates identified between the three research databases. The initial identification of records identified in the three research databases are as follows:

- Academic Search Complete: 468,278;
- Complementary Index: 429,021;
- MEDLINE Complete: 308,457.

A full-text review of the 118 articles identified upon completion of database screening by the research team resulted in an additional 76 articles being excluded from the review. These articles were removed from the review for the following reasons:

- Primary focus of mental distress and/or mental health was not observed;
- Letters to the editor;
- Additional duplicates;
- The article was not directly related to the COVID-19 pandemic.

The research team conducted article reviews using the article assignment shown in Table 1. Each article was analyzed by at least two of the research team members (or more) and consensus meetings were held via online webinar. A MS Excel spreadsheet was kept to gather all observations by the research team and ultimately develop underlying constructors (facilitators and barriers) regarding the research topic. The team experienced no differences of opinion or any other dissent toward both any article and the underlying concept(s) identified in the final review articles.

### 3.2. Study Characteristics

A systematic approach was employed in reviewing articles to determine the protective and non-protective factors of mental health distress in the United States during the COVID-19 pandemic. In addition to the Johns Hopkins Nursing Evidence-Based Practice (JHNEBP) study design analysis, both protective and non-protective factors of mental distress during the pandemic are summarized in Table 2. Articles are listed in alphabetical order by the first author’s last name.

### 3.3. Risk of Bias

The research team utilized the JHNEBP quality indicator frequencies to assess the strength of evidence of all articles included in the review. These frequencies are shown in Table 3. As with any review, a higher frequency in levels I and/or II categories is preferred, although, often, this is not always a possibility based upon the review topic and other related variables included in the search. Based upon this review’s initiative to investigate those studies surrounding protective and non-protective factors of mental health distress within a specific timeframe (COVID-19 pandemic), and also based within the unique U.S. healthcare system, articles with JHNEBP quality indictor levels I through IV were identified by the review team. Level V articles were not permitted in the study primarily due to the opinion and/or letter to the editor status (addressed in Figure 1).

This review served as a convenience sample in an attempt to assess mental health protective and non-protective factors of mental health distress within the unique U.S. healthcare system. As a result, the study does not review identified constructs beyond the U.S., and results beyond this one country are limited with regard to external validity (to some extent).

### 3.4. Additional Analysis

**Figure 2:**
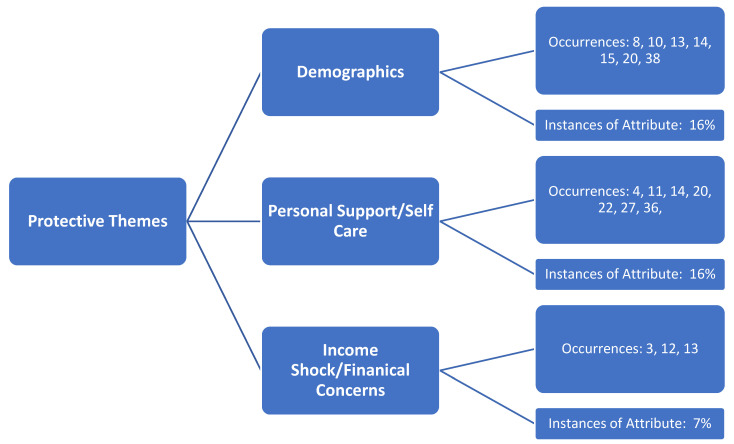
Identified themes (constructs) identified as protective factors of mental health distress during the COVID-19 pandemic in the United States.

**Figure 3:**
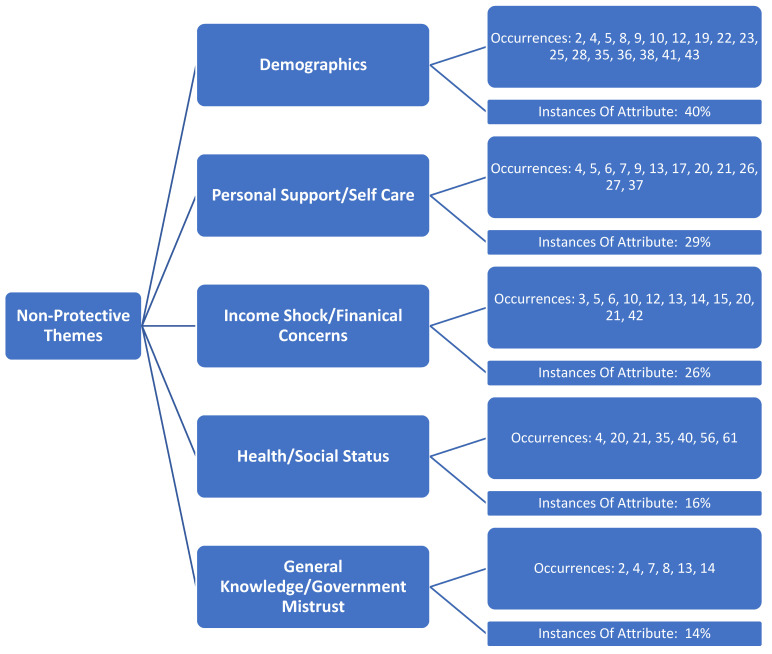
Identified themes (constructs) identified non-protective factors of mental health distress during the COVID-19 pandemic in the United States.

**Figure 4:**
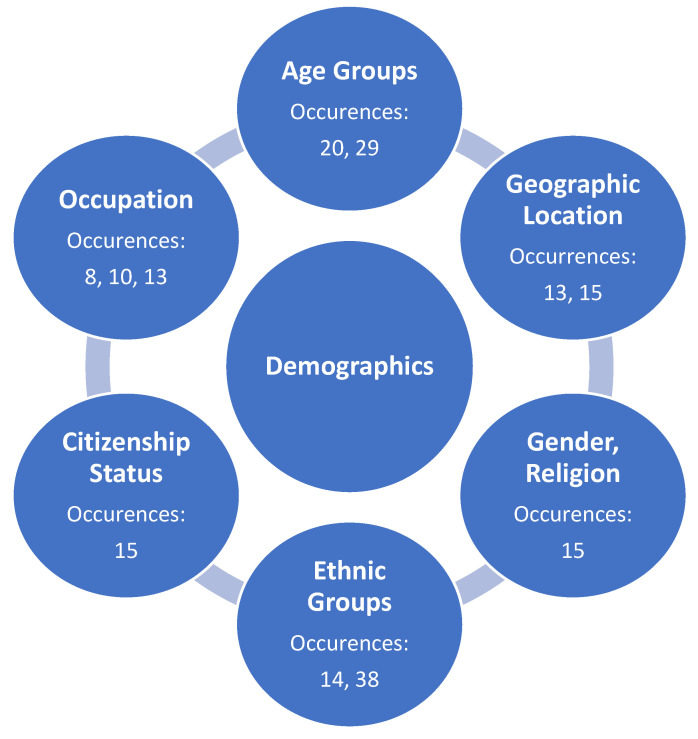
Identified sub-themes (underlying sub-constructs) identified protective demographic factors of mental health distress during the COVID-19 pandemic in the United States.

**Figure 5:**
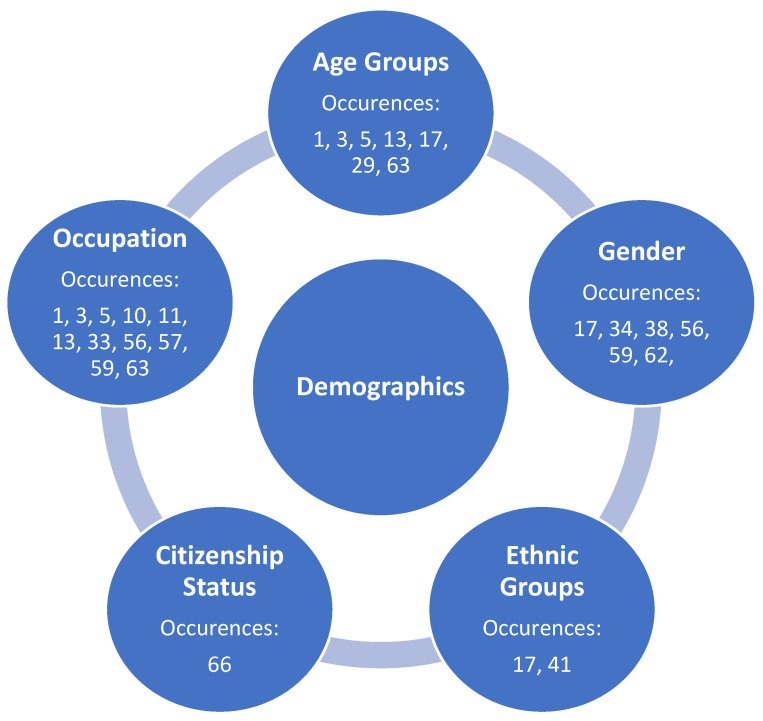
Identified sub-themes (underlying sub-constructs) identified non-protective demographic factors of mental health distress during the COVID-19 pandemic in the United States.

Identified underlying themes (constructs) by the review team are shown in Figure 2 and Figure 3. Three protective factors were identified (Figure 2), and these same three constructs were also considered as non-protective factors, along with two additional non-protective constructs (Figure 3). The review team concluded that the three matching constructs among both protective and non-protective mental health distress observed in the literature were not easily related and/or had a dichotomous relationship and, therefore, were to be presented separately as protective and non-protective factors. Further, findings in Figure 2, Figure 3, Figure 4 and Figure 5 are not mutually exclusive to either theme, and, as a result, several articles demonstrate more than one construct upon review.

The research team initially identified various stakeholder characteristics related to demographics that demonstrated protection against mental health distress as the COVID-19 pandemic continues. This protective theme (construct) was created as a result of the collapsing of multiple sub-variable categories originally established by the review team on the affinity matrix, included within Figure 4.

The research team also identified various stakeholder characteristics related to demographics that demonstrated non-protective factors against mental health distress as the COVID-19 pandemic continues. This non-protective theme (construct) was created as a result of the collapsing of multiple sub-variable categories originally established by the review team on the affinity matrix, included within Figure 5.

## 4. Discussion

### 4.1. Summary of Evidence

The pandemic has resulted in an increased prevalence of mental health distress among the population of the United States. This review surveyed the literature and identified both protective and non-protective variables associated with mental health distress in the United States during the COVID-19 era. As a result, several underlying constructs for both protective and non-protective factors were identified by the research team.

Three main underlying themes were identified in the literature by the research team that supported protective factors towards mental health distress in the U.S. (Figure 2). Demographics occurred in the literature at a rate of 16% of the total articles in the review. Personal support/self-care (16% occurrence) and income shock/financial concerns (7% occurrence) were also able to be identified, and articles were classified under each of these constructs, as identified by the team.

The research team identified three primary themes (constructs) associated with both non-protective factors associated with mental health distress during the pandemic. Non-protective variables identified were demographics (40% occurrence), personal support/self-care (29% occurrence), income shock/financial concerns (26% occurrence), health/social status (16% occurrence), and general knowledge/government mistrust (14% occurrence). Within-construct sub-variables regarding protective and non-protective factors associated with mental health distress during the pandemic were also able to be identified by the research team, and are discussed in their respective section(s) below.

### 4.2. Protective Theme: Demographics

For instance, Caucasians were identified in the literature as having less depression than other races [14,38]. Age was also an influencing factor on less mental distress, with both older (above 65 years old) and traditional college-age students identified in this category [20]. While citizenship [15], gender [15], religion [15], and occupation were also identified by the review team in this vein, results also indicate that individuals living in states with a lower prevalence of COVID-19, as well as rural areas, demonstrate less mental distress from the pandemic [13,15]. Additional research is required to further identify both these and other potential demographic indicators of mental health distress as being related to COVID-19 in order to further strengthen this review construct.

### 4.3. Protective Theme: Personal Support and Care

During the systematic literature review, several factors have been grouped into a larger category, which is coined “personal support and care”. Although demographic factors are addressed separately in this review, demographic classification, such as age, is an important distinction with the factors in this category. Having robust social networks and connections proved to be protective for all ages for mental health distress during the pandemic [4,20,26,35]. Paradoxically, a subset of young adults with underlying social anxiety disorder perceived less stress with the recommended social distancing practices. Nursing students who had effective family functioning and spiritual support experienced less mental distress during the pandemic [22]. The quality of the family relationship was found to be particularly protective in Hispanic youth and adolescents [38]. The removal from in-house school activities is postulated to increase the quality of family relationships and increase self-care behaviors, such as sleep, yet relieve youth from peer stressors prevalent in adolescence [30]. Nurses caring for COVID-19 patients reported self-care practices, such as meditation, psychological support, exercise, and sleep hygiene, to be especially important is staving off mental health distress [17].

College age students and young adults’ coping mechanisms to the pandemic, such as spiritual practices, developing a daily routine, practicing positive reframing, the use of social media and streaming services, journaling, music, reading, drawing, time with pets, and increasing physical activity, all proved to be protective [22,36]. Older adults’ protective coping mechanisms included self-reflection, reliance on memories, and reflections of life experiences [27]. Spending more time outside in daylight was protective for all ages [20]. One study examined three factors of religious variables in the American Orthodox Jewish population: intrinsic religiosity, religious coping, and trust in God, and found all three variables to be protective factors to mental health distress during the pandemic [31]. A separate study showed practice of religiosity in young American adults (age 18–29), as well as a conservative political affiliation, to also be protective [15]. Lastly, based on research about these practices in the absence of a global pandemic, adults practicing yoga, mindfulness, and healthy living habits experience less mental distress; therefore, it is suggested that this benefit would carry over as being protective during the pandemic [4].

### 4.4. Protective Factor: Income Shock and Financial Concerns

The COVID-19 pandemic has had a massive economic impact in the U.S., leading to the largest one-quarter economic contraction since record keeping began in 1945 [3]. During times of economic volatility, the provision of unemployment insurance can serve as an important safety net, and the research team confirmed protective variables associated with this benefit on mental health during the pandemic. Unemployment insurance was shown to mitigate the risk of mental health distress by helping people continue to meet health-related social needs, such as food and housing, and reducing depressive and anxiety symptoms [3]. This literature review validated the health benefits of unemployment insurance through the identification of protective factors associated with states that offered greater maximum unemployment insurance and/or states with more weeks of unemployment insurance. These states, in comparison to others with less generous unemployment benefits for those undergoing income shock and financial distress, showed less deleterious effects on mental health during the pandemic [3,12,13].

In addition to variation in state-level policies related to unemployment benefits during the pandemic, the researchers found that other protective policies to support citizens experiencing income shock lessened psychological distress. For example, states that implemented a moratorium on evictions or utility shutoffs and had previously expanded Medicaid eligibility under the Affordable Care Act showed different experiences of mental health related to income shocks during the pandemic [12]. This systematic review strongly confirms that households experiencing an income shock during the pandemic were less distressed if they happen to live in states with social policies that reduce economic insecurity and ensure access to health care.

### 4.5. Non-Protective Theme: Demographics

Compared to the frequency of occurrences identified in the review as protective factors related to demographics (nine identified articles), non-protective factors falling within this same construct were much higher (19 identified articles). This difference suggests that demographics play a more influential role in contributing to mental health distress during the pandemic, as opposed to protecting against related distress. Additional research is required to further identify specific demographic characteristics and within-category relationship interactions of this review construct.

The following sub-variables were identified by the review team and collapsed into the non-protective demographic variable (Figure 5):

Occupation was identified by the review team as a significant non-protective demographic factor of mental distress during the pandemic, most often related to close-contact healthcare-related jobs, such as nursing and dental hygiene [2,8,22,35]. Further, those positions with ongoing COVID-19 exposure, specifically prone to airborne diseases, were identified [8,22].

### 4.6. Non-Protective Theme: Lack of Personal Support and Care

In the personal support and care protective factor section, social support, including family, friends, and social networks, and the quality of such relationships, were key protective factors. As such, the absence of this support network causing social isolation or a history of family traumas are significant causes of mental distress in all age groups [3,5,6,13,15,17,20,21,36]. College students and older adults alike had increased depressive and suicidal thoughts due to the feelings of loneliness, insecurity, and hopelessness brought on by social isolation [28,36]. College students related the following barriers to mitigating the psychological distress with counseling services to include a lack of insight into the severity of their symptoms, not being comfortable talking to strangers or on the phone about their concerns, and a lack of trust and discomfort in accessing school mental health services [36]. A different type of isolation negatively impacted nurses whose extensive isolation caused by their professional requirements further isolated them from family, friends, community, and political groups, causing increase mental health distress [17].

College students and adults using avoidant coping mechanisms, such as denial or disengagement, experienced increased mental health distress during the pandemic [27,36]. The use of alcohol as a coping mechanism was non-protective, and one study showed that alcohol use in the U.S. increased during the first wave of the pandemic in states with, interestingly, a lower burden of COVID-19 cases [20,26]. Unhealthy eating habits and the “pandemic baking syndrome” increased as coping mechanisms or as a product of “stay at home” and “social distancing” requirements, and led to an increase in eating disorders [6,36]. Having more liberal political beliefs and experiencing spiritual struggles were nonprotective factors experienced in US adults [15,31]. Although social connection appears to be a protective factor, having interpersonal or social media communication with COVID-19 specific content increases psychological distress in those who had had direct exposure with COVID-19 (personally infected, hospitalized, or experienced death of a loved one from COVID-19) [14].

### 4.7. Non-Protective Theme: Income Shock/Financial Concerns

The tremendous job loss and wage cuts during the COVID-19 pandemic has raised concerns about the mental health of the population, considering the well-established findings that financial stressors are leading contributors to suicide, substance abuse, and other mental health issues. As discussed in our section on protective factors, states with different policy contexts likely influence mental health. This systematic review verifiably determined that the lack of unemployment insurance—or a lessor benefit to insulate from the shock of lost household income—is a key non-protective variable [3]. However, a non-protective thematic review goes much deeper, in that there are a wider breadth of sub-variables identified. The associations of the increase in psychological distress with income shock may be reinforced by gender, age, ethnicity, or vulnerability.

There are a constellation of factors that contribute to the worsening of mental health during the pandemic (e.g., infection concerns, social isolation), but income shock as experienced by low income households was the most significant non-protective sub-variable identified in correlation [10,21,42]. Age was also a significant non-protective theme identified by our research group. Given that young adults were disproportionately more likely to work in sectors of the economy that were shut down during the pandemic (e.g., retail, restaurants, leisure facilities), the associated financial insecurity and job loss experienced by many young people contributed to a sustained rise in depression levels [10,12,15]. The research team found that households that experienced an income shock were more likely to experience heightened levels of depression and anxiety if they were non-white, had lower levels of educational attainment, and were divorced or never married [12].

Another noteworthy non-protective sub-variable associated with income shock and mental health distress was geographic setting, as unemployment insurance and other government aid during the pandemic was shown to mitigate psychological symptoms for those primarily in non-urban areas [13]. In summary, our research group found in the systematic review of the literature that a rise in unemployment during the pandemic is associated with significantly elevated mental health problems, and that several non-protective sub-variables exist in this relationship.

### 4.8. Non-Protective Theme: Baseline Health/Social Status

Individuals experienced high levels of distress for a countless number of reasons throughout the course of the COVID-19 pandemic; two of which include preexisting medical illnesses and an unstable income as a result of a low socioeconomic status [20,21,42]. Those with preexisting health conditions, particularly mental health conditions, experienced higher levels of fear than their respective counterparts [28]. However, a combination of the two underlying health and socioeconomic status reasons revealed the most significant effect on mental health [28,35,40].

### 4.9. Non-Protective Theme: Lack of Knowledge and/or Lack of Government Trust

Throughout the country, there has been uncertainty surrounding the pandemic and inconsistent or culturally incompetent messaging. This has added to an increase in fear and a general lack of knowledge about the COVID-19 pandemic [2,4,13,14]. The polarized politicized environment and increased focus on social justice has further contributed to an increased burden on mental health [7,8].

## 5. Study Limitations

As with any systematic literature review, limitations do exist. While the review identified 42 manuscripts, only 9 articles (22%) were categorized by the research team as JHNEBP Level I (experimental study/randomized control trial). Additionally, 29 articles (70%) were classified as Level II (quasi-experimental studies), and the remaining 8% of the review articles fell within Level III (non-experimental, qualitative) and Level IV (opinion of nationally recognized experts based on research evidence/consensus panels). As a result, the research team had to utilize broader-level themes to categorize underlying constructs. Finally, this review focused on mental health and related mental distress protective and non-protective factors that were specifically identified within the U.S. This exclusion criteria was applied to the database to help narrow results to a more manageable level for the research team, while also helping to describe a single country’s protective and non-protective factors for mental health distress during the pandemic. While exclusive to only the U.S., findings may be appliable to other countries with similar environmental, economic, and governmental conditions, and this remains a strong area for future study.

## 6. Conclusions

This systematic review identified protective and non-protective themes regarding the effects on mental health in the nation during COVID-19. The findings suggest that factors such as occupation, financial uncertainty, and a lack of social support result in an increase in mental health distress among individuals. Healthcare students and workers experience high levels of distress relative to their counterparts. Individuals with previously diagnosed health problems experienced higher levels of mental distress. While these protective and non-protective themes vary by specific factors, the study suggests self-care, a steady income, and a strong support system positively affected mental health during the pandemic. Identified facilitators and barriers identified are directly influenced by the United States health care system, and this study suggests challenges and best practices offered by U.S. outpatient organizations that may also be beneficial for other countries.

## Figures and Tables

**Table 1:** Reviewer assignment of the initial database search findings (full article review).

Article Assignment	Articles 1–11	Articles 12–22	Articles 23–33	Articles 34–44	Articles 45–55	Articles 56–66
Reviewer 1	X			X		
Reviewer 2	X	X		X	X	
Reviewer 3		X	X		X	X
Reviewer 4			X			X
Reviewer 5			X			X

Upon completion of the review, a total of 42 articles were included in the study.

**Table 2:** Summary of findings (n = 42).

Author(s)	Participant(s)	* JHNEBP Study Design	Protective Factors of Mental Health Distress during COVID-19	Non-Protective Factors of Mental Health Distress during COVID-19
Arnetz et al. [2]	U.S. (Michigan) nurses	II	Less direct exposure to COVID patients. Survey mental health among nurses and proactively identify those in high-risk groups and in need of support.	Lack of access to adequate personal protective equipment (PPE) led to increased reporting of symptoms of depression, anxiety, and post-traumatic stress disorder. Lack of overall emergency preparedness led to front-line nurses being exposed to unprecedented stress. Witnessing risk of infection by colleagues getting sick/dying, seeing patients die alone.
Berkowitz and Basu [3]	Analysis of U.S. Census Bureau’s Household Pulse Survey public use files	II	Being in a home receiving unemployment insurance benefits was associated with fewer health-related social needs and better mental health.	The lower benefit levels received by unemployment insurance beneficiaries after the expiration of Federal Pandemic Unemployment Compensation (FPUC) were associated with greater risk for unmet health-related social needs and worse mental health.
Bhattacharjee and Acharya [4]	Review article	II	Preventing close contact with other individuals through the use of social media for healthy communications with family members and friends. Developing a supportive network where people may share each other’s worries and discuss strategies. Focusing on the positives and improving skills to establish professional marketability at the individual level. Regulation of eating/sleeping habits and performing yoga/meditation.	Population groups susceptible to mental health illnesses include elderly people, health care professionals, professionals (non-health care with exposure to COVID-19), children/teenagers, and people with prior psychiatric history. Inadequate knowledge leads to misinformation and unnecessary panic among the people.
Breslau et al. [5]	National probability sample of adults in the United States.	I	n/a	Hispanics were more likely to report an increase in psychological distress than other racial/ethnic groups. Distress may be driven more by economic stressors than fears specific to the disease, since older individuals are widely reported to be at higher risk of morbidity and mortality related to the virus. Finding of higher risk among women is consistent with prior studies of psychiatric disorders following disasters. Social distress prior to COVID-19 was highly related to distress during the pandemic.
Chee et al. [6]	People living in Canada or the United States	II	n/a	Reduced hours and being laid off were associated with greater stress appraisals, avoidant- and emotion-focused coping responses, and negative effects. Some coping strategies may contribute to the greater vulnerability to downstream effects, particularly those relating to eating choices and nutritional balances.
Christensen et al. [7]	Cross-sectional survey of 1030 U.S. adults	II	n/a	Females and those with lower income experienced more COVID-19 related economic anxieties. Those working and with children at home reported higher social, home, and work disruption. Social distancing behaviors were more common among liberals and were associated with increases in depressive symptoms.
Cipriano et al. [8]	American Nursing Association (ANA) report on membership.	IV	n/a	Social injustice, personal loss, a contentious Presidential election, and inability to control the virus compound the stress and burnout of nurses and other healthcare workers, creating serious mental health consequences. Those with preexisting mental health challenges were at greater risk for burnout and even suicide.
Comfort et al. [9]	Outpatient reproductive health providers across the U.S. engaged in contraceptive care.	II	n/a	Many providers reporting stress, anxiety or depression mentioned changes in job responsibilities, with several examples of providers managing testing sites. Anxiety and depression centered around inadequate PPE, fear of coming to work, and fear of getting sick or getting family members sick. Financial concerns and childcare responsibilities.
Daly et al. [10]	Participants were recruited via address-based sampling using the US Postal Service Computerized Delivery Sequence file covering almost 100% of US households.	I	Statistically significant increases in depression levels were observed for all population subgroups examined, with the exception of those aged 65+ years and Black participants.	n/a
Długosz, P. [11]	Probability-based online panel of adults living in households in the United States	I	Males, people living in relationships, practicing religion more often, having a better financial situation, conservative beliefs, and being devoid of citizenship had a better mental condition.	Highest levels of mental discomfort have been observed among the youngest Americans aged 18–29.
Donnelly and Farina [12]	2020 Household Pulse Survey (U.S.)	I	Living in a state with supportive social policies—primarily those related to Medicaid, unemployment insurance, and suspended utility shut offs during the pandemic—weakens the association between household income shocks and mental health.	Prevalence of depression and anxiety differs across states by household income shock status.
Fan and Nie [13]	Nationally representative sample of adults aged 18 and above in the United States and a regional representative sample of adults aged 18 and older living in 18 different geographic areas, including 10 states and 8 metropolitan statistical areas (MSAs).	I	AA working-age group experiences similar or more favorable mental health than other ethnic groups.	Government aid only mitigates the psychological symptoms for the group in non-urban areas, with no significant impacts on the urban group. Government aid does not alleviate the mental pressure for the AA group.
First et al. [14]	Adults (18 years or older) living in the United States.	II	COVID-19 had an indirect effect on stress and depression through media use (traditional and social) and interpersonal communication.	COVID-19 exposure had a direct effect on stress.
Hidalgo et al. [15]	Cross-sectional study used data collected during the first wave of the COVID-19 Adult Resilience Experiences Study (CARES)2020 Project	II	n/a	Young adults experienced high rates of sleep problems during the first two months (April to May2020) of the pandemic. Depressive and anxiety symptoms appear to be predictors of sleep quality, regardless of any pre-existing diagnosis. High levels of post-traumatic stress disorder (PTSD) symptoms and COVID-19-related worry were associated with young adults’ poor sleep.
Hyun et al. [16]	Convenience sample of registered nurses working in an acute care setting or in units with diagnosed COVID-19 patients.	III	n/a	Acute care nurses working with limited access to PPE during the COVID-19 pandemic.
Iheduru-Anderson [17]	18 years or above and an approved foster carer in the participating US state convenience sample.	II	Age, financial security, and mental health status were the strongest determinants of post-care practices.	Foster careers who were married, not employed outside their home, reported good mental and physical health, and were financially stable exhibited higher levels of self-care compared to their peers.
Miller and Grise-Owens [18]	Longitudinal cohort study using the COVID-19 Adult Resilience Experiences Study.	II	n/a	Sexual and gender minority young adults had significantly higher levels of depression and PTSD symptoms, as well as COVID-19-related worries and grief, than non-SGM (sexual and gender minority), even after controlling for family support, lifetime discrimination, and pre-existing mental health diagnoses.
Kamal et al. [19]	Representative sample of 1013 U.S. adults	II	Getting outside more often, perceived social support, and older age were protective against these problems.	Prevalence estimates were 1.5–1.7 times higher for those who reported job losses due to COVID-19 restrictions. Mental health problems were predicted by worry over financial instability, insomnia, social isolation, and alcohol consumption.
Kilgore et al. [20]	U.S. COVID-19 Household Impact Survey.	I	n/a	Experiencing COVID-19 restrictions significantly raises mental distress. Association is stronger for individuals with preexisting health conditions and those who worry about job prospects.
Kim and Laurence [21]	Cross-sectional online survey was conducted from 20 April to 10 May 2020 among 173 nursing students at a private university in Southern California, USA.	II	High levels of resilience and family functioning were associated with 2- to 2.4-fold lower risk of stress, anxiety, and depression in nursing students. High spiritual support was associated with two-fold lower risk of depression for nursing students.	Nursing students’ self-reported stress, anxiety, and depression were significantly higher during the lockdown compared to the pre-lockdown period.
Kim et al. [22]	Adult pregnant and post-partum (up to 6 months postdelivery) women in April–June2020 in the United States.	I	Pervasive uncertainty and anxiety; grief about losses; gratitude for shifting priorities; and use of self-care methods, including changing media use.	The most common predictors were job insecurity, family concerns, eating comfort foods, resilience/adaptability score, sleep, and use of social and news media.
Kinser et al. [23]	Large representative sample of the adult population of Spain.	II	n/a	Higher number of women were affected than men and a greater increase was observed in younger people.
Le and Nguyen [24]	A national sample of English-speaking women aged 18 years was recruited from a continuously refreshed research panel maintained by Opinions 4 Good (Op4G), a survey research firm	II	n/a	Odds of depression, anxiety, and posttraumatic stress symptoms were two to three times higher among women who reported at least one incident or worsening health-related socioeconomic risk.
Lindau et al. [25]	Self-reported data from the Understanding America Study (UAS), a national, longitudinal survey	II	n/a	Individuals living in states with higher COVID-19 burdens reported a higher average number of drinking days at the beginning of the epidemic. As the pandemic progressed, respondents living in states with lower COVID-19 burdens increased the number of drinking days throughout the first wave of the pandemic. The increases in alcohol consumption were exclusively among those living in states with a relatively low disease burden, whereas those living in states with a relatively high burden did not increase alcohol consumption frequency.
McKetta et al. [26]	Participants between the ages of 18–92 were recruited from social media posts and ResearchMatch, an online research registry connecting participants with institutional Review-Board-approved studies	II	Age was negatively related to posttraumatic stress, each mental health outcome, and avoidant coping, such that older individuals were less stressed, had better psychosocial functioning, and were less likely to use avoidant coping behaviors.	Posttraumatic stress was highly correlated with the psychosocial outcome variables of depression, anxiety, and loneliness in the expected direction. Posttraumatic stress was also associated with the proposed mediators of coping style (avoidant and approach) and social support in the expected direction.
Minahan et al. [27]	Utilized data from the pandemic in the United States (and informed by data from other countries), as well as past theorizing and empirical research on the views and treatment of older adults.	IV	Positive responses can reinforce the value of older adults, improve older adults’ mental and physical health, reduce ageism, and improve intergenerational relations, whereas negative responses can have the opposite effects.	Social distancing to protect older adults from COVID-19 infection) can inadvertently increase loneliness, depression, health problems, and negative stereotyping of older adults.
Monahan et al. [28]	Participants from American School Health Association membership list were contacted via electronic mail.	II	n/a	Wellness factors (mental health, physical education, and activity) have a long history of being secondary to academic priorities. The COVID-19 pandemic may worsen existing mental health problems and lead to more cases among children and adolescents as internal and external factors, such as social isolation and economic recession, worsen.
Pattison et al. [29]	Individuals 10 to 14 years of age in grades 5 to 8 who were attending a public charter middle school in a large city in southwestern United States	II	A significant reduction in mental health problems for youths who had elevated levels of internalizing, attention, externalizing, or total problems before the pandemic from baseline to follow-up 1, while controlling for age and gender. Being removed from the in-person school environment led to improved mental health due to a reduction in peer stressors. Academic pressures may also have been reduced once in-person school was closed. Lack of in-person schooling led to more flexible routines that allowed for adolescents to receive more sleep.	n/a
Penner et al. [30]	Participants resided in the USA and identified with an Orthodox Jewish religious affiliation	II	Strong evidence for positive impact of the pandemic. These findings may attest to general human resilience in the face of trauma. Results may reflect unique resilience related to religious coping. Positive religious coping, intrinsic religiosity, and trust in God emerged as strong correlates of less stress and increased positive impact, as previous research suggests.	Fear of exposure to COVID-19 was related to negative coping and mistrust of God, and negative religious coping and mistrust in God correlated with increased stress and less positive impact.
Turchioe et al. [31]	Cross- sectional study in late March 2020 with a national sample of 963 US adults.	II	n/a	Female, younger generations, and financial resources have been associated with worse mental health symptoms.
Reppas-Rindisbacher et al. [32]	U.S. and Canada adults over 55 years old.	II	n/a	U.S. older adults felt less supported by their federal government and had elevated depressive and anxiety symptoms compared to older adults in Canada during early months of the COVID-19 pandemic.
Robinson and Daly [33]	Probability-based longitudinal study of 9063 adults recruited using address-based sampling from the US Postal Service Computerized Delivery Sequence file covering almost 100% of US households.	I	Personal health concerns reduced, as did financial concerns, and changes in lifestyle because of COVID-19 became less likely, which all mediated the decrease in psychological distress.	Personal health concerns (perceived risk of infection and mortality from COVID-19) rose sharply, and these concerns accounted for a substantial amount of the initial rise in distress. Perceived financial risks (i.e., running out of money) and changes in lifestyle characterized by reductions in social contact also increased and explained 14–15% of the initial rise in distress.
Rollins [34]	Electronically distributed survey was sent to all United States-based pediatric anesthesiology fellowship program directors, who were asked to distribute the survey to all current/graduating fellows	II	n/a	A majority of respondents experienced increased stressors during this pandemic, including worry for family members, stress due to changes in certifying examinations, and fear of contracting COVID-19 from a patient.
Son et al. [35]	Students at a large public university in the United States	II	Almost half of the participants reported lower stress levels related to academic pressure and class workload since the pandemic began.	Difficulty in concentrating, frequently expressed by our participants, has previously been shown to adversely affect students’ confidence in themselves [29], which has known correlations to increased stress and mental health. 44% of the participants reported experiencing an increased level of depressive thoughts, and 8% reported having suicidal thoughts associated with the COVID-19 pandemic. The majority of our participants exhibited maladaptive coping behaviors.
Szilagyi and Olezeski [36]	Case study analysis for transgender youth	III	Community support, strong group identification, and family affirmation can serve as important mitigating factors. Virtual visits have the potential to interfere with development of a therapeutic alliance and the movement toward increased family acceptance.	n/a
Thomaier et al. [37]	United States cancer-care physicians	II	n/a	Demographic factors associated with anxiety included female sex, young age, and less time in clinical practice. Perception of inadequate personal protective equipment and practicing in a state with more COVID-19 cases were associated with anxiety symptoms. Factors significantly associated with both anxiety and depression included the degree to which COVID-19 has interfered with the ability to provide treatment to cancer patients, and concern that patients will not receive the level of care needed for non-COVID-19 illness.
Valdez et al. [38]	English language US tweets collected from an open-access public repository	II	Aggregated social media feeds are shown to adequately predict other phenomena, including the stock market; political leanings; and, when analyzed through a timeseries, collective shifts in general mood.	Social media content is reactionary to news cycles. Study found a negative trajectory in sentiment scores for the user timeline data.
Vidot et al. [39]	Adults 18 years old or older who self-reported medicinal cannabis use within the past year	I	n/a	Over half of adults who use medicinal cannabis reported fear of giving COVID-19 to someone else or fear of being diagnosed.
Wade et al. [40]	Prospective cohort of 549 caregivers designed to understand the effects of COVID-19 stress and disruption on family wellbeing	II	n/a	Female caregivers are, on average, considerably more burdened than male caregivers in terms of their experience of COVID stress and their self-reported history of childhood adversity. Female caregivers report significantly more mental health problems than male caregivers in the domains of distress, anxiety, and posttraumatic stress.
Wang et al. [41]	U.S. residents recruited through the panel provider Qualtrics for a larger longitudinal study about the effects of COVID-19	II	n/a	Anxiety symptoms and greater job insecurity due to COVID-19 were related to greater depressive symptoms. Greater financial concern was related to greater anxiety symptoms.
Xu et al. [42]	Qualtrics Panels of U.S. residents to collect cross-sectional survey data from grandparent kinship providers	II	Caregivers with better physical health might be more sensitive to feeling increased parenting stress, particularly during these uncertain times. If a grandchild has better mental health, grandparents would be less stressed than those that have a child with worse mental health.	Grandparents’ mental health distress is associated with increased parenting stress.
Yarrington et al. [43]	Data collected via Youper, a mental health app	II	People find ways to cope with life-changing negative circumstances. Habituation to or reductions in anxiety are common in cases of prolonged exposure.	Women drove both an initial increase and subsequent decrease in anxiety compared to other genders. Full-time employees drove declines in optimism.

* Johns Hopkins Nursing Evidence-Based Practice (JHNEBP) levels of strength of evidence: Level I, experimental study/randomized control trial (RCT). Level II, quasi-experimental study. Level III, non-experimental, qualitative, or meta-synthesis study. Level IV, opinion of nationally recognized experts based on research evidence/consensus panels. Level V, opinions of industry experts not based on research evidence.

**Table 3:** Summary of quality assessments.

Strength of Evidence	Frequency
I (Experimental/RCT)	9 (22%)
II (Quasi-experimental)	29 (70%)
III (Non-experimental, qualitative)	2 (4%)
IV (Opinion of nationally recognized experts based on research evidence/consensus panels)	2 (4%)
